# Time Trends in Spain from 2001 to 2018 in the Incidence and Outcomes of Hospitalization for Urinary Tract Infections in Patients with Type 2 Diabetes Mellitus

**DOI:** 10.3390/ijerph17249427

**Published:** 2020-12-16

**Authors:** Ana López-de-Andrés, Romana Albaladejo-Vicente, Domingo Palacios-Ceña, David Carabantes-Alarcon, José Javier Zamorano-Leon, Javier de Miguel-Diez, Marta Lopez-Herranz, Rodrigo Jiménez-García

**Affiliations:** 1 Department of Public Health & Maternal and Child Health, Faculty of Medicine, Universidad Complutense de Madrid, 28040 Madrid, Spain; anailo04@ucm.es (A.L.-d.-A.); dcaraban@ucm.es (D.C.-A.); josejzam@ucm.es (J.J.Z.-L.); rodrijim@ucm.es (R.J.-G.); 2 Department of Physical Therapy, Occupational Therapy, Rehabilitation and Physical Medicine, Rey Juan Carlos University, Alcorcón, 28922 Madrid, Spain; domingo.palacios@urjc.es; 3 Respiratory Department, Hospital General Universitario Gregorio Marañón, Instituto de Investigación Sanitaria Gregorio Marañón (IiSGM), 28009 Madrid, Spain; javier.miguel@salud.madrid.org; 4 Department of Medicine, Faculty of Medicine, Universidad Complutense de Madrid, 28040 Madrid, Spain; 5 Faculty of Nursing, Physiotherapy and Podology, Universidad Complutense de Madrid, 28040 Madrid, Spain; martal11@ucm.es

**Keywords:** urinary tract infections, type 2 diabetes mellitus, incidence, trends, mortality

## Abstract

We aim to examine the incidences, clinical characteristics, and in-hospital outcomes of type 2 diabetes (T2DM) patients hospitalized with urinary tract infections (UTIs) in Spain and to identify the factors associated with in-hospital mortality (IHM). A retrospective observational study was carried out with a sample that included all adult patients who were hospitalized for UTIs between 2001 and 2018 and collected in the Spanish National Health System Hospital Discharge Database. We identified 850,276 patients with UTIs (25.49% with T2DM). The incidence of UTIs increased in patients with and without diabetes from 290.76 and 74.79 cases per 100,000 inhabitants in the period from year 2001 to year 2003 to 568.45 and 144.0 in the period from 2016 to 2018, respectively (*p* < 0.001). Adjusted incidence of UTIs was higher in T2DM patients (incidence rate ratio (IRR) 4.36; 95% CI 4.35–4.39). The multivariable analysis showed a significant reduction in the IHM over time for men and women with T2DM. In T2DM, patients’ higher IHM was associated with older age, comorbidities, and *Staphylococcus aureus* isolation. Women with T2DM had a higher risk of dying than men. The risk of IHM with an episode of UTIs was independent of the presence of T2DM (odds ratio (OR) 0.97; 95% CI 0.91–1.01). We conclude that the incidence of UTIs was over four times higher in T2DM than nondiabetic patients and has increased over time.

## 1. Introduction

Urinary tract infections (UTIs) are the most common type of bacterial infections in the community and, also, at hospital settings, resulting in high rates of morbidity and high economic costs associated with its treatment [1]. Diabetes is a complex disease that is characterized by a state of chronic hyperglycemia and is often associated with the increasing risk of several infectious diseases, including UTIs [2,3].

Several studies have reported that UTIs are more frequent in patients with type 2 diabetes mellitus (T2DM) than in the general population [4,5]. Hirji et al. [5] concluded that the risk of UTIs was 53% greater among T2DM patients compared with a matched cohort of nondiabetic patients. Nichols et al. [6] also found that T2DM was associated with an adjusted 25% increased risk of UTI (rate ratio 1.25, 95% CI 1.22–1.29). Although the exact mechanism is unknown, several possibilities have been proposed to explain the association between diabetes and UTI. Patient-related factors that were found to enhance the risk for UTI in diabetics include age, metabolic control, and long-term complications, primarily diabetic nephropathy and cystopathy [7]. On the other hand, it is less clear whether clinically modifiable outcomes like, for example, glycated hemoglobin (HbA1C), or patient characteristics like the body mass index (BMI) may have their own predictive role with regards to the UTI risk in T2DM patients [8,9].

The increased risk of UTI among diabetic patients, coupled with the increase in the incidence of T2DM worldwide in recent years, may impose a substantial burden on medical costs in the next decades [10]. To the best of our knowledge, data on the incidence of UTIs in patients with and without diabetes have not been reported in the Spanish population. Our hypothesis is that the incidence of UTIs has possibly increased over time among T2DM patients, who suffer these infections with a higher frequency and worse hospital outcomes than nondiabetic patients. Therefore, in this study, we used national hospital discharge data to examine trends in incidence and outcomes of UTIs among men and women with and without T2DM in Spain from 2001 to 2018. In particular, we analyzed patient comorbidities, procedures, UTI pathogens, and in-hospital outcomes, such as in-hospital mortality (IHM) and length of hospital stay (LOHS). Finally, we identified the predictors for IHM after hospitalization with UTIs among men and women with T2DM.

## 2. Materials and Methods

### 2.1. Study Design and Data Collection

We conducted a retrospective observational study using the Spanish National Hospital Discharge Database (SNHDD). We included all hospital admissions between 1 January 2001 and 31 December 2018. The SNHDD is an administrative database that collects deidentified demographic, clinical, and resource utilization data of all public and private Spanish hospitals. Every year, over 95% of Spanish hospitals send their data to the Ministry of Health, which freely provides the requested databases to investigators [11].

The principal and secondary diagnoses and the therapeutic and diagnosis procedures conducted during hospital admission are codified using the International Classification of Diseases (ICD). From 2001 to 2015, the SNHDD used the 9th Revision Clinical Modification (ICD-9-CM) that was replaced by the 10th Revision (ICD-10) from 2016 onwards. Details of the database can be found elsewhere [11].

We selected admissions for patients aged 18 or over with a primary diagnosis of UTIs based on the definition of the Agency for Healthcare Research and Quality (AHRQ) Prevention Quality Indicator 12 for Urinary Tract Infections [12]. As the SNHDD is anonymized, it is possible that the same patient is included more than once along the study period, because these duplications cannot be detected.

### 2.2. Study Variables

We grouped admissions by diabetes status as follows: T2DM (ICD-9-CM codes: 250.x0 and 250.x2 and ICD-10: E11.x) or no diabetes in any diagnostic position. We excluded people with type 1 diabetes mellitus (ICD-9-CM codes: 250.x1 and 250.x3 and ICD-10: E10.x).

The study outcomes variables are the incidence of UTIs per 100,000 inhabitants and in-hospital variables such as the LOHS and IHM.

We calculated the yearly T2DM-specific incidence rates by dividing the number of admissions per year, sex, and age group by the corresponding number of people in that population group using the age-adjusted, sex-adjusted estimated prevalence of T2DM obtained from National Health Surveys (NHS) conducted in years 2001/2002, 2003/2004, 2006/2007, 2009/2010, 2011/2012, 2014/2015, and 2016/2017 and based on data from the Di@bet.es Study, which estimated the prevalence of diabetes in the Spanish population [13,14]. Diabetic populations for the missing years (2005, 2008, 2013, and 2018) were estimated assuming that the growth rate was the same through the period. We also calculated the yearly, age, and sex-adjusted-specific incidence rates for nondiabetic patients by dividing the number of cases per year, sex, and age group by the corresponding number of people in that population group (excluding those with T2DM), according to the data from the Spanish National Institute of Statistics, as reported on 1 January of each year [15].

IHM is defined by the proportion of patients who died during admission for each year of the study.

For each hospital admission, we analyzed the sex and age of the patients as demographic variables. To describe the comorbid conditions that might alter the risk of mortality, we used the Charlson Comorbidity Index (CCI) using the algorisms for administrative databases using the ICD 9 and ICD 10 codes described by Quan et al. [16].

They also analyzed the presence of isolated microorganisms that was assessed using the ICD codes shown in Supplementary Table S1. We specifically identified patients with codes for *Enterococcus*, *Staphylococcus aureus*, *Klebsiella pneumoniae*, *Escherichia coli*, *Proteus*, and *Pseudomonas aeruginosa* in any diagnosis field.

According to the SNHDD, only pathogens that have been laboratory-confirmed can be included in the database [11]. The variable “urinary catheter” was created using the procedure codes described in Supplementary Table S1. The time period from 2001 to 2018 was analyzed in six three-year periods to fit in the descriptive tables.

### 2.3. Statistical Methods

Our investigation was conducted using a sex-stratified analysis. The reason for this is that previous investigations documented sex differences in lower urinary tract anatomy, biology, and physiology that explain the significant differences in the epidemiology, etiology, clinical course, and treatments of UTIs between men and women [17,18,19].

Poisson regression models adjusted by age were used to assess the time trends for study groups providing incidence rate ratios (IRR) with 95% confidence intervals as the measure of association.

Sample characteristics were described using proportions for categorical variables and mean and standard deviation (SD) or median and interquartile range (IQR) for continuous variables. To compare proportions, we used the chi-square test, Student’s *t*-test for means, and Wilcoxon–Mann-Whitney Test for medians.

Time trend for study variables was assessed using bivariate logistic regression (proportions), ANOVA (means), or the Kruskal-Wallis test (medians), as appropriate.

The Bonferroni correction for multiple comparisons was used to control the familywise error rate (FWER). The critical value (alpha) for an individual test is obtained by dividing the FWER (0.05) by the number of tests in the “family” of statistical tests conducted. In each table, the number of tests that the *p*-value should be corrected by is indicated in the footnote [20,21].

A multivariable logistic regression model was constructed to identify the predictors of IHM among T2DM patients with UTIs providing odds ratios (ORs) with 95% CI. Finally, using the entire database, we analyzed the effect of T2DM on the IHM.

To conduct the multivariable regression models, the following steps were done: (i) A univariate analysis of each variable was performed. (ii) Variables for the multivariable analysis were selected by including all the variables that were significant in the univariate analysis and those we considered scientifically relevant according to the references reviewed. (iii) In order to fit the multivariable model, the importance of each variable included in the model was verified. This included examining the Wald statistic. (iv) Once the model was obtained, we more closely evaluated the included variables (linearity). Finally, we checked for interactions in the model. All multivariable analyses were constructed using time as a continuous variable.

The variables in the final models, including confounders, are listed as footnotes in the tables.

Stata version 14 (Stata, College Station, Texas, USA) was used for data analysis. Commands used in STATA for Poisson and Logistic regression were “*poisson*” and “*logic*”, respectively.

### 2.4. Ethical Aspects

According to the Spanish legislation, as we used the SNHDD, which is a deidentified retrospective public access database that is provided freely to all investigators by the Spanish Ministry of Health, it was not necessary to obtain approval by an ethics committee or informed consent by the patients.

## 3. Results

We identified a total of 850,276 hospitalizations of patients with a primary diagnosis of UTIs (25.49% with T2DM) in Spain between 2001 and 2018. The prevalence of T2DM was 20.09% in 2001/2003, increasing to 26.67% in the period 2016/18 (*p* < 0.001). In patients who had an admission for UTIs, there was a significant female predominance (59.1% in T2DM and 59.5% in the nondiabetes population). Among those suffering T2DM hospitalized with UTIs, the mean age rose three years among men and five years among women from 2001 to 2018 (*p* < 0.001). The proportion of men withT2DM aged 85 years or over increased from 15.36% in the period 2001/3 to 24.51% (*p* < 0.001) in the last period. Among women, the equivalent increment was from 20.88% to 40.08% (*p* < 0.001).

### 3.1. Time Trends in the Incdence of UTIs According to T2DM Status

The detailed incidence rates per 100,000 inhabitants according to the diabetes status, age groups, and sex in Spain from 2001 to 2018 are shown in Table 1.

Among patients with T2DM, we found that the incidence of UTIs coding increased significantly from 290.76 in 2001/2003 to 568.45 cases per 100,000 inhabitants in 2016/2018 (*p* < 0.001). In patients without T2DM, the incidence of admissions also increased significantly over the study period from 74.79 to 144.0 cases per 100,000 inhabitants (*p* < 0.001).

The incidence of UTIs coding increased both in men and women with T2DM significantly from 228.68 and 344.32 cases per 100,000 T2DM population in 2001/2003 to 474.02 and 672.33 in 2016/2018, respectively (all *p* < 0.001). The equivalent increase in the figures for men and women without T2DM patients were found (Table 1).

The incidence was significantly higher in women than in men for all years analyzed and besides diabetes status. Over the entire time period, the incidence in T2DM women was 546.5 and in T2DM men was 376.69 (*p* < 0.001). Among T2DM patients, overall and in men and women separately, the incidence increased with age in all time periods. For the entire time period, the incidence was highest among men and women aged ≥85 years, with incidence rates per 100,000 inhabitants of 1640 and 1980, respectively.

The results of the Poisson regression models showed that the overall incidence of UTIs over the period 2001–2018 was 4.36 times higher among patients with T2DM than among those without T2DM (IRR 4.36; 95% CI 4.35–4.39). Additionally, these models show equivalent figures for men and women with T2DM (men: IRR 4.24; 95% CI 4.21–1.71 and women: IRR 4.49; 95% CI 4.47–4.53) when compared to nondiabetic men and women. Shown in Supplementary Figure S1 are the IRR plotted for each time period for men and women with T2DM compared with nondiabetic T2DM men and women. As can be seen in this figure, the IRR for both sex IRRs were stable and ranged between 3.6 and 5.

### 3.2. Time Trends in the Characteristics of UTIs According to T2DM Status

The clinical characteristics and hospital outcomes for admissions of T2DM patients with a principal diagnosis of UTIs in Spain from 2001 to 2018 are shown in Table 2.

Overall, men with T2DM were older (75.39; SD = 10.83 years) than patients without diabetes (70.19; SD = 16.85 years; *p* < 0.05) and had more coexisting medical conditions (mean CCI index: 1.07 ± 0.77 vs. 0.85 ± 0.71). Age, CCI, and use of a urinary catheter increased significantly over time in both men with and without T2DM.

Regarding hospital outcomes, the median LOHS was six days in men with and without T2DM and remained stable overtime. For the total time period, crude IHM was higher in men with T2DM than nondiabetic men (4.57% vs. 4.33%; *p* < 0.001), and IHM decreased significantly from 5.55% and 4.52% in 2001/3 for men with and without T2DM to 4.17% and 4.19% in 2016/18, respectively (both *p* < 0.001).

Among women, the overall T2DM patients were older (78.28 vs. 63.88 years), had higher CCI (mean CCI: 0.89 vs.0.58), and had more frequency of urinary catheters (3.56% vs. 2.75%) during UTI admissions than women without T2DM. In both groups, women increased their mean age, their mean CCI, and the frequency of urinary catheters over the study period (all *p* < 0.001). The median LOHS was significantly higher in women with T2DM (six vs. five days). Over time, the LOHS falls significantly in those with T2DM. For the total time period, the crude IHM was 5.18% for women with T2DM and 3.75% for nondiabetic women (*p* < 0.001). The IHM tended to be stable over time in women with T2DM, though, in women without T2DM, increased over time.

When we compared T2DM patients according to sex, we found that women were older, had lower CCI, had lower use of a urinary catheter, and had higher crude IHM than men (all comparisons *p* < 0.001), as can been seen in Table 2.

The isolated pathogens among patients according to diabetes status and sex are shown in Table 3.

Among men, the most frequently isolated pathogens were *Escherichia coli* (T2DM: 25.6% and non-T2DM: 25.07%), *Klebsiella pneumoniae* (T2DM: 5.92% and non-T2DM: 4.76%), and *Pseudomonas aeruginosa* (T2DM: 5.24% and non-T2DM: 5.65%).

The most prevalent pathogens isolated among women were *Escherichia coli* (T2DM: 34% and non-T2DM: 32.46%), *Klebsiella pneumoniae* (T2DM: 5.89% and non-T2DM: 3.63%), and *Enterococcus* (T2DM: 2.89% and non-T2DM: 2.43%). In all groups and both sexes, the prevalence of all the pathogens analyzed increased significantly over time.

The age distribution, comorbidities, procedures, and in-hospital outcomes among men and women hospitalized with a principal diagnosis of urinary tract infection in Spain, 2001–2018, according to the presence of isolated pathogens and T2DM status, are shown in Supplementary Tables S3 and S4. As can be seen in these tables, beside the sex and T2DM status, having an isolated pathogen was associated with a lower IHM (all *p* < 0.001).

### 3.3. Variables Associated with IHM among Men and Women with UTIs According to T2DM Status

The variables associated with IHM after a multivariable analysis in patients with T2DM are shown in Table 4.

For both sexes, the risk of dying increased with age and with suffering a higher number of conditions included in the CCI.

The insertion of a urinary catheter was a protective factor among men (OR 0.69; 95% CI 0.6–78) and a risk factor for women (OR 1.2; 95% CI 1.07–1.36).

Regarding isolated pathogens, *Escherichia coli* and *Klebsiella pneumoniae* were associated with a lower risk of dying during hospitalization. However, the isolation of *Staphylococcus aureus* increased the risk of IHM by 40% among men (OR 1.4; 95% CI 1.19–1.66) and 82% among women (OR 1.82; 95% CI 1.57–2.13).

After adjusting by study variables, the result of the multivariable analysis showed a significant reduction in the IHM over time for men and women with T2DM.

When possible, confounders were controlled; T2DM women had a higher risk of dying during their hospitalization the men (OR 1.1; 95% CI 1.05–1.14).

Lastly, in our study, after adjusting for study variables, suffering T2DM was not associated with IHM in people admitted to the hospital with an episode of UTI (OR 0.97; 95% CI 0.91–1.01 for the total population, OR 0.95; 95% CI 0.90–1.02 for men, and OR 0.99; 95% CI 0.93–1.04 for women), as can been seen in Supplementary Table S2.

## 4. Discussion

This population-based study showed that the overall incidence rate of hospitalization for UTIs in patients with T2DM has doubled over the 18-year period. Furthermore, the adjustment for potential confounders showed that the independent effects of T2DM on the incidence of discharge remained significant for both sexes. The incidence of UTI observed in our study was consistent with the findings of other authors [5,6]. One large study of 135,920 patients with T2DM and a 1:1 matched group without diabetes in the UK General Practice Research Database reported crude UTI incidence rates of 46.9/1000 patients per year and 29.9/1000 patients per year for patients with and without T2DM [5]. Recently, in a US study using annual data from the National Health Interview Survey, it found that, in 2015, the rates of hospitalization with an UTI remained more than four times as high in adults with vs. without diabetes (RR 4.3 (95% CI 4.3–4.4)) [22], results very similar to ours.

In the present study, the rates of hospitalization for UTIs increased in patients with T2DM from 2001 to 2018. Over a comparable study period between 2000 and 2015, the population surveillance conducted in the USA detected increasing rates of hospitalization for this infection type in people with diabetes [22]. However, in Hong Kong, a recently published study found that UTIs remained unchanged from 2001 to 2016 in patients with diabetes and concluded that it is possible that the increasing tendency to treat less severe UTIs in an outpatient setting could contribute to that stabilization [23]. Differences in the health services organizations could explain these different trends. Additionally, changes in the treatment of T2DM practice over time, especially the introduction of SGLT2 inhibitors in 2012 by the European Medicines Agency, might be another reason for the increased incidence of UTIs in the diabetes mellitus cohort [24,25].

In addition to a higher overall UTI rate, we found that age and female gender were positively associated with UTI risk, results consistent with the existing literature [19]. A large observational study of UTIs in older adults (aged ≥65 years) conducted from 2004 to 2014 in the UK showed that, in women, the incidences increased from 9–11 cases per 100 people per year in subjects aged 65–74 years to 11.4–14.3 cases and 14.7–19.8 cases per 100 people per year in subjects aged 75–84 and >84 years, respectively. The corresponding values in men were 2.8–3.0, 5.9–6.1, and 8.1–10.5 cases per 100 people per -year [26]. Diabetic elderly women are thought to be at increased risk for UTIs, presumably due to immunological and metabolic changes associated to neurological abnormalities secondary to diabetes [27]. Glycosuria and the increased formation of advanced glycosylation end products may play a role in the development of diabetic complications and may also contribute to the development of UTIs, because these factors can lead to disturbances in monocyte migration and cytokine production [2,28,29].

Our results document that women and men with T2DM were older and had higher CCI than those without diabetes, consistent with the findings of previous studies [5,22,23]. However, even if age and comorbidities increased among T2DN patients, the IHM decreased over time in both sexes. Possible reasons for this improvement include better medical initiatives and organization of care that has led to improved glycemic control and the prevention of infections requiring hospitalization in adults with diabetes [22,30].

Regarding the pathogens isolated, *E. coli* was the most frequent infectious agent among T2DM patients with UTIs and the number of patients with a diagnosis a pathogen isolation increased over time. It has been suggested that the increase in a rapid molecular identification of *E. coli* at the sub-strain level, as well as the prediction of antibiotic resistances, might enable a more efficient selection of antibiotics for treatment and, in part, reduce the role of this pathogen over time [31].

As we expected, older patients and a higher CCI were the variables most closely associated with IHM for women and men with T2DM. Laudisio et al. [32] indicated that the CCI is a predictor of mortality in patients hospitalized for UTI, and polymicrobial UTIs are more common in older populations, and they are associated with increased disease severity.

A catheter-associated urinary tract infection can increase the length of patients’ stays, cost of patient care, and mortality. When difficult urethral catheterization does occur, the catheterization emergency can easily escalate out of control, leading to acute urethral catheterization injury with bleeding requiring hospital admission for more invasive specialist procedures [33,34]. We found that the insertion of a urinary catheter was a risk factor of IHM in women; however, in men, it was a protective factor. Given the limited information collected by the SNHDD, the association between a urinary catheter and IHM may be due to factors not controlled in this study and should be interpreted with caution.

Although the exact relationship between catheter-associated bacteriuria and mortality is uncertain, the populations at the highest risk of mortality include women, elderly patients, and immunosuppressed patients [35,36].

The female sex was a risk factor for mortality in T2DM patients with UTIs. Previous studies have postulated several explanations for these results. In our study, women were older than men in both patients with and without diabetes. Advanced age is a well-known risk factor for UTIs in T2DM women. Sewify et al. [37] concluded that diabetes is associated with a higher risk of acute symptomatic UTI in postmenopausal women than younger women.

We described a positive decline in IHM overtime in both genders. In a recent study, Greeg et al. [30] analyzed trends in age-specific death rates and proportional mortality from all causes, including UTIs, among US adults by diabetes status from 1988–1994 to 2010–2015 and found reductions in all of them. The authors concluded the improvement in diabetes control and UTI diagnosis, and treatment over time could justify this trend [30].

After a multivariable adjustment, T2DM was not identified as a factor associated with IHM among men and women after UTIs. A Greek study of 225 patients hospitalized with acute pyelonephritis included diabetes among 13 potential risk factors extracted from a chart review for the analysis of the outcomes of mortality or prolonged hospitalization. In the multivariate analysis, diabetes mellitus had an odds ratio of 5.3 (*p* < 0.01) for women and 4.7 for men (*p* < 0.001) for prolonged hospitalization but was not associated with the increased mortality [38].

In our investigation, an increased risk of IHM was associated with UTI if *Staphylococcus aureus* was found. It has been previously described that, in up to 34% of cases, *Staphylococcus aureus* bacteriuria is associated with bacteremia by this microorganism. These patients frequently have a complicated course with higher hospital mortality [39].

Sewify et al. [37] indicated that most of the UTI cases (78.2%) were found in the diabetic patients with uncontrolled glycemia. The authors concluded that accurate screening for UTIs in diabetic patients is also critical to enable the appropriate treatment, avoiding related complications.

The results of this study have some implications for public health. In this study, we show that diabetes confers an almost four-fold increased risk for UTI-related hospitalization. The increasing number of people living with diabetes is likely to increase the number of people with UTIs in the future and will have important implications for hospital burdens and patient care. Furthermore, improved awareness by healthcare providers that diabetes is an important risk factor for UTIs might improve an early diagnosis and treatment. For example, the assessment of diabetes at hospital admission for an UTI may help physicians more effectively manage the glucose levels.

There are some points that should be taken into consideration when interpreting the results of the present study. Our data source was the SNHDD, an administrative database that contains discharge data for hospitalizations in Spain and uses information the physician included in the discharge report [11]. Coding practices, as well as errors in coding, may differ between individual physicians and institutions. Thus, our results are subject to several potential biases, including differences in the capture of adverse outcomes across hospitals or even a diabetes diagnosis during the study period. Another limitation that should be considered is changes in the coding practices over time.

According to the methods used, it is possible that the same person may be hospitalized more than once along the study period. In any case, as our objective is to assess the magnitude of the effect of T2DM in the incidence and consequences of UTI hospitalizations in Spain, in our opinion, from an epidemiological point of view, it is not relevant if a UTI was suffered by the same or a different patient. If T2DM increases the risk of suffering UTIs, it is logical that people with T2DM suffer a higher number of ITU hospitalizations in their lifetime than non-T2DM patients, and this will be shown in the analysis only if we include all hospital admissions besides if they are or are not in a duplicated patient.

Our findings are limited by the lack of data of glycosylated hemoglobin measurements and did not have the blood glucose levels to evaluate the degree of control of diabetes during admissions; also, data on diabetes duration or treatment is not available in the database. Other studies have identified factors that may influence UTI outcomes and that were not included in our investigation, because these variables were not collected in the SNHDD. These factors include, among others, the moment of acquisition of the UTIs or antimicrobial treatments.

Despite these limitations, our national estimates are based on a large sample size, the 18-year follow-up period, and the standardized methodology, which has been used to investigate other infections in Spain and elsewhere [40].

## 5. Conclusions

Our study reveals that the incidence of UTIs was higher in T2DM patients than in those without this disease and increased over time in both groups of patients and in both sexes. The IHM decreased over time in both men and women with T2DM, despite a concomitant increase in UTI diagnoses during the same period. Higher mortality rates in T2DM patients were associated with the female sex, age, presence of more comorbidities, and a diagnosis of *Staphylococcus aureus* isolation. T2DM was not associated with the IHM after multivariable adjustments. These results suggest that the management of UTIs has improved in Spain during the study period among T2DM patients. Future investigations are necessary to identify preventive programs, protocols, and interventions that can help to prevent and mitigate this burdensome complication.

## Figures and Tables

**Table 1:** Hospital admissions among patients with a principal diagnosis of urinary tract infection per 100,000 inhabitants in Spain from 2001 to 2018, according to diabetes status, age groups, and sex.

		2001–2003	2004–2006	2007–2009	2010–2012	2013–2015	2016–2018	Total	*p*-Value
Sex and T2DM Status	Age Groups	N (Inc/10^5^)	N (Inc/10^5^)	N (Inc/10^5^)	N (Inc/10^5^)	N (Inc/10^5^)	N (Inc/10^5^)	N (Inc/10^5^)	
Men with T2DM	18–50 years	241 (53.32)	289 (63.17)	323 (82.59)	368 (73.58)	364 (69.92)	371 (79.38)	1956 (70.14)	<0.001
	51–64 years	959 (102.35)	1370 (107.98)	1750 (131.53)	2247 (134.78)	2705 (183.68)	2804 (171.35)	11,835 (142.38)	<0.001
	65–74 years	1980 (235.01)	2593 (266.47)	3207 (325.62)	3798 (367.67)	5021 (402.59)	5982 (374.61)	22,581 (338.16)	<0.001
	75–84 years	2438 (417.67)	3634 (583.5)	5319 (711.17)	6647 (763.61)	7644 (939.85)	8636 (873.54)	34,318 (741.72)	<0.001
	≥85 years	1012 (1203.7)	1387 (984.83)	2155 (1343.5)	3260 (1702.1)	4208 (1866.33)	5776 (2041.47)	17,798 (1640)	<0.001
	Total	6630 (228.68)	9273 (267.77)	12,754 (352.82)	16,320 (382.89)	19,942 (466.02)	23,569 (474.02)	88,488 (376.69)	<0.001
Men without T2DM	18–50 years	5525 (17.8)	5627 (16.97)	5835 (17.64)	5462 (17.15)	5839 (18.99)	6124 (21.29)	34,412 (18.24)	<0.001
	51–64 years	5069 (57.47)	5919 (62.15)	6848 (65.84)	7413 (69.11)	8923 (77.64)	10,436 (83.89)	44,608 (70.36)	<0.001
	65–74 years	6682 (150.79)	6938 (171.06)	7617 (179.7)	7960 (178.01)	10,041 (212.03)	12,305 (261.71)	51,543 (193.52)	<0.001
	75–84 years	8052 (341.91)	9653 (330.44)	11,681 (415.15)	13,205 (481.58)	14,674 (532.74)	16,587 (603.67)	73,852 (452.13)	<0.001
	≥85 years	4292 (741.14)	5151 (886.31)	7238 (951.87)	9012 (1083.19)	11,196 (1179.62)	14,942 (1580.61)	51,831 (1115.33)	<0.001
	Total	29,620 (62.71)	33,288 (66.26)	39,219 (76.45)	43,052 (85.04)	50,673 (100)	60,394 (121.76)	256,246 (85.51)	<0.001
Women with T2DM	18–50 years	384 (128.91)	457 (95.63)	552 (124.1)	592 (100.29)	702 (130.46)	554 (122.48)	3241 (115.7)	0.051
	51–64 years	1422 (166.42)	1631 (200.2)	1847 (195.1)	2024 (193.36)	2171 (226.18)	2157 (236.08)	11,252 (203.25)	<0.001
	65–74 years	2980 (257.76)	3446 (340.54)	3587 (351.89)	3909 (336.76)	4259 (392.01)	4426 (340.5)	22,607 (335.69)	<0.001
	75–84 years	4370 (528.85)	6126 (664.64)	7921 (728.75)	9917 (878.85)	10,742 (1052.16)	11,073 (825.6)	50,149 (792.8)	<0.001
	≥85 years	2416 (1068.86)	3430 (1556.48)	5451 (1800.7)	7868 (2035.54)	9660 (2289.12)	12,179 (2374.55)	41,004 (1980.35)	<0.001
	Total	11,572 (344.32)	15,090 (437.83)	19,358 (509.36)	24,310 (563.68)	27,534 (683.68)	30,389 (672.33)	128,253 (546.5)	<0.001
Women without T2DM	18–50 years	16,364 (53.87)	19,136 (60.86)	20,386 (64.36)	19,795 (63.13)	21,227 (70.77)	21,459 (75.16)	118,367 (64.54)	<0.001
	51–64 years	5138 (54.85)	5607 (53.33)	6224 (55.61)	6964 (60.53)	8474 (68.26)	9931 (73.96)	42,338 (61.88)	<0.001
	65–74 years	5697 (103.47)	5879 (106.81)	5779 (112.53)	6411 (123.57)	7558 (132.68)	8968 (157.97)	40,292 (123.19)	<0.001
	75–84 years	8780 (271.59)	10,613 (273.62)	12,638 (316.95)	14,886 (364.93)	16,625 (418.09)	18,717 (494.52)	82,259 (358.59)	<0.001
	≥85 years	6769 (645.52)	8406 (884.42)	12,520 (876.79)	16,562 (1088.1)	21,066 (1215.15)	28,710 (1542.77)	94,033 (1100.62)	<0.001
	Total	42,748 (86.3)	49,641 (94.93)	57,547 (107.73)	64,618 (120.45)	74,950 (139.27)	87,785 (164.69)	377,289 (119.39)	<0.001

T2DM: Type 2 Diabetes Mellitus. Inc/10^5^: incidence per 100,000 inhabitants. *p*-values for the time trend using Poisson regression analysis adjusted by age. According to the Bonferroni correction for multiple comparisons, the critical value (alpha) should be divided by 6 (0.05/6 = 0.0083).

**Table 2:** Comorbidities, procedures, and in-hospital outcomes among patients with a principal diagnosis of urinary tract infections in Spain, 2001–2018, according to diabetes status and sex.

Sex and T2DM Status	Variables	2001–2003	2004–2006	2007–2009	2010–2012	2013–2015	2016–2018	Total	*p*-Value
Men with T2DM	Age, mean (SD)	73.54 (11.26)	74 (11.03)	74.96 (10.74)	75.39 (10.85)	75.65 (10.77)	76.49 (10.58)	75.39 (10.83)	<0.001
	CCI mean (SD)	0.89 (0.71)	0.97 (0.75)	1.01 (0.73)	1.06 (0.74)	1.07 (0.76)	1.21 (0.84)	1.07 (0.77)	<0.001
	CCI = 0	2594 (39.13)	3352 (36.15)	4338 (34.01)	5077 (31.11)	6234 (31.26)	6594 (27.98)	28,189 (31.86)	<0.001
	CCI 1–2	2619 (39.5)	3619 (39.03)	4994 (39.16)	6541 (40.08)	7846 (39.34)	8889 (37.71)	34,508 (39)	
	CCI > 2	1417 (21.37)	2302 (24.82)	3422 (26.83)	4702 (28.81)	5862 (29.4)	8086 (34.31)	25,791 (29.15)	
	Urinary catheter, *n* (%)	349 (5.26)	532 (5.74)	920 (7.21)	1299 (7.96)	1800 (9.03)	1904 (8.08)	6804 (7.69)	<0.001
	LOHS, median (IQR)	6 (7)	7 (7)	6 (6)	6 (6)	6 (6)	6 (6)	6 (5)	0.454
	IHM, *n* (%)	368 (5.55)	508 (5.48)	675 (5.29)	743 (4.55)	766 (3.84)	984 (4.17)	4044 (4.57)	<0.001
Men without T2DM	Age, mean (SD)	66.86 (17.99)	68 (17.55)	69.42 (17.2)	70.78 (16.57)	71.28 (16.23)	72.2 (15.9)	70.19 (16.85)	<0.001
	CCI mean (SD)	0.68 (0.61)	0.76 (0.67)	0.8 (0.68)	0.89 (0.71)	0.91 (0.72)	0.95 (0.78)	0.85 (0.71)	<0.001
	CCI = 0	14,782 (49.91)	15,570 (46.77)	17,383 (44.32)	17,278 (40.13)	19,794 (39.06)	23,635 (39.13)	108,442 (42.32)	<0.001
	CCI 1–2	10,542 (35.59)	11,915 (35.79)	14,267 (36.38)	16,232 (37.7)	19,308 (38.1)	21,999 (36.43)	94,263 (36.79)	
	CCI > 2	4296 (14.5)	5803 (17.43)	7569 (19.3)	9542 (22.16)	11,571 (22.83)	14,760 (24.44)	53,541 (20.89)	
	Urinary catheter, *n* (%)	1365 (4.61)	1823 (5.48)	2842 (7.25)	3386 (7.86)	4648 (9.17)	4612 (7.64)	18,676 (7.29)	<0.001
	LOHS, median (IQR)	6 (7)	7 (7)	6 (6)	6 (6)	6 (6)	6 (6)	6 (5)	0.214
	IHM, *n* (%)	1339 (4.52)	1456 (4.37)	1863 (4.75)	1899 (4.41)	2002 (3.95)	2532 (4.19)	11,091 (4.33)	<0.001
Women with T2DM	Age, mean (SD)	75.03 (11.83)	76.17 (11.41)	77.49 (11.45)	78.56 (11.19)	79.04 (11.27)	80.15 (10.85)	78.28 (11.36)	<0.001
	CCI mean (SD)	0.68 (0.59)	0.74 (0.63)	0.82 (0.64)	0.88 (0.66)	0.92 (0.68)	1.07 (0.66)	0.89 (0.69)	<0.001
	CCI = 0	5722 (49.45)	6943 (46.01)	8055 (41.61)	9396 (38.65)	10,166 (36.92)	9598 (31.58)	49,880 (38.89)	<0.001
	CCI 1–2	4216 (36.43)	5632 (37.32)	7596 (39.24)	9718 (39.98)	11,055 (40.15)	11,975 (39.41)	50,192 (39.14)	
	CCI > 2	1634 (14.12)	2515 (16.67)	3707 (19.15)	5196 (21.37)	6313 (22.93)	8816 (29.01)	28,181 (21.97)	
	Urinary catheter, *n* (%)	226 (1.95)	384 (2.54)	622 (3.21)	929 (3.82)	1261 (4.58)	1144 (3.76)	4566 (3.56)	<0.001
	LOHS, median (IQR)	7 (7)	7 (7)	6 (7)	6 (6)	6 (5)	6 (5)	6 (6)	<0.001
	IHM, *n* (%)	601 (5.19)	847 (5.61)	1109 (5.73)	1245 (5.12)	1292 (4.69)	1549 (5.1)	6643 (5.18)	0.062
Women without T2DM	Age, mean (SD)	58.75 (23.94)	59.12 (24.29)	61.58 (24.44)	64.52 (23.83)	65.9 (23.4)	68.36 (22.85)	63.88 (23.95)	<0.001
	CCI mean (SD)	0.42 (0.36)	0.45 (0.3)	0.51 (0.44)	0.6 (0.49)	0.65 (0.52)	0.72 (0.57)	0.58 (0.49)	<0.001
	CCI = 0	28,493 (66.65)	32,331 (65.13)	35,344 (61.42)	36,155 (55.95)	39,931 (53.28)	43,547 (49.61)	215,801 (57.2)	<0.001
	CCI 1–2	11,197 (26.19)	13,080 (26.35)	16,327 (28.37)	20,066 (31.05)	23,877 (31.86)	29,336 (33.42)	113,883 (30.18)	
	CCI > 2	3058 (7.15)	4230 (8.52)	5876 (10.21)	8397 (12.99)	11,142 (14.87)	14,902 (16.98)	47,605 (12.62)	
	Urinary catheter, *n* (%)	585 (1.37)	873 (1.76)	1478 (2.57)	2024 (3.13)	2798 (3.73)	2621 (2.99)	10,379 (2.75)	<0.001
	LOHS, median (IQR)	5 (6)	5 (5)	5 (5)	5 (5)	5 (5)	5 (5)	5 (5)	<0.196
	IHM, *n* (%)	1466 (3.43)	1653 (3.33)	2184 (3.8)	2426 (3.75)	2745 (3.66)	3670 (4.18)	14,144 (3.75)	<0.001

T2DM: Type 2 Diabetes Mellitus. CCI: Charlson Comorbidity Index. LOHS: Length of Hospital Stay. SD: Standard deviation. IQR: Interquartile Range. IHM: In-Hospital Mortality. *p*-value < 0.05 to assess the time trend from 2001 to 2018. Using the χ^2^ test for linear trends (proportions) and ANOVA (means) as appropriate. According to the Bonferroni correction for multiple comparisons, the critical value (alpha) should be divided by 6 (0.05/6 = 0.0083).

**Table 3:** Isolated pathogens codified in hospital admissions among patients with a principal diagnosis of urinary tract infection in Spain, 2001–2018, according to diabetes status and sex.

Sex and T2DM Status	Variables	2001–2003	2004–2006	2007–2009	2010–2012	2013–2015	2016–2018	Total	*p*-Value
Men with T2DM	*Enterococcus*, *n* (%)	179 (2.7)	264 (2.85)	443 (3.47)	732 (4.49)	1095 (5.49)	1401 (5.94)	4114 (4.65)	<0.001
	*Staphylococcus aureus*, *n* (%)	174 (2.62)	275 (2.97)	366 (2.87)	423 (2.59)	525 (2.63)	612 (2.6)	2375 (2.68)	0.305
	*Klebsiella pneumoniae*, *n* (%)	137 (2.07)	264 (2.85)	433 (3.4)	775 (4.75)	1469 (7.37)	2163 (9.18)	5241 (5.92)	<0.001
	*Escherichia coli*, *n* (%)	1254 (18.91)	2058 (22.19)	2906 (22.79)	4338 (26.58)	5615 (28.16)	6486 (27.52)	22,657 (25.6)	<0.001
	*Proteus*, *n* (%)	139 (2.1)	197 (2.12)	330 (2.59)	406 (2.49)	652 (3.27)	804 (3.41)	2528 (2.86)	<0.001
	*Pseudomonas aeruginosa*, *n* (%)	213 (3.21)	383 (4.13)	584 (4.58)	933 (5.72)	1185 (5.94)	1339 (5.68)	4637 (5.24)	<0.001
Men without T2DM	*Enterococcus*, *n* (%)	733 (2.47)	963 (2.89)	1341 (3.42)	1876 (4.36)	2985 (5.89)	3631 (6.01)	11,529 (4.5)	<0.001
	*Staphylococcus aureus*, *n* (%)	645 (2.18)	805 (2.42)	1036 (2.64)	1161 (2.7)	1319 (2.6)	1480 (2.45)	6446 (2.52)	<0.001
	*Klebsiella pneumoniae*, *n* (%)	471 (1.59)	696 (2.09)	1157 (2.95)	1809 (4.2)	3195 (6.31)	4864 (8.05)	12,192 (4.76)	<0.001
	*Escherichia coli*, *n* (%)	5677 (19.17)	6912 (20.76)	9020 (23)	11,330 (26.32)	14,172 (27.97)	17,135 (28.37)	64,246 (25.07)	<0.001
	*Proteus*, *n* (%)	576 (1.94)	702 (2.11)	956 (2.44)	1164 (2.7)	1544 (3.05)	2048 (3.39)	6990 (2.73)	<0.001
	*Pseudomonas aeruginosa*, *n* (%)	1172 (3.96)	1513 (4.55)	2072 (5.28)	2666 (6.19)	3413 (6.74)	3644 (6.03)	14,480 (5.65)	<0.001
Women with T2DM	*Enterococcus*, *n* (%)	169 (1.46)	307 (2.03)	462 (2.39)	726 (2.99)	895 (3.25)	1150 (3.78)	3709 (2.89)	<0.001
	*Staphylococcus aureus*, *n* (%)	182 (1.57)	262 (1.74)	372 (1.92)	405 (1.67)	446 (1.62)	436 (1.43)	2103 (1.64)	0.002
	*Klebsiella pneumoniae*, *n* (%)	238 (2.06)	462 (3.06)	744 (3.84)	1281 (5.27)	1949 (7.08)	2877 (9.47)	7551 (5.89)	<0.001
	*Escherichia coli*, *n* (%)	3228 (27.89)	4614 (30.58)	6003 (31.01)	8309 (34.18)	10,097 (36.67)	11,358 (37.38)	43,609 (34)	<0.001
	*Proteus*, *n* (%)	248 (2.14)	300 (1.99)	481 (2.48)	573 (2.36)	776 (2.82)	815 (2.68)	3193 (2.49)	<0.001
	*Pseudomonas aeruginosa*, *n* (%)	169 (1.46)	257 (1.7)	382 (1.97)	572 (2.35)	631 (2.29)	664 (2.19)	2675 (2.09)	<0.001
Women without T2DM	*Enterococcus*, *n* (%)	476 (1.11)	676 (1.36)	1099 (1.91)	1534 (2.37)	2330 (3.11)	3067 (3.49)	9182 (2.43)	<0.001
	*Staphylococcus aureus*, *n* (%)	486 (1.14)	603 (1.21)	829 (1.44)	892 (1.38)	1068 (1.42)	1065 (1.21)	4943 (1.31)	<0.001
	*Klebsiella pneumoniae*, *n* (%)	551 (1.29)	840 (1.69)	1326 (2.3)	2097 (3.25)	3616 (4.82)	5279 (6.01)	13,709 (3.63)	<0.001
	*Escherichia coli*, *n* (%)	10,844 (25.37)	13,210 (26.61)	16,638 (28.91)	21,574 (33.39)	27,433 (36.6)	32,755 (37.31)	122,454 (32.46)	<0.001
	*Proteus*, *n* (%)	742 (1.74)	888 (1.79)	1136 (1.97)	1557 (2.41)	1926 (2.57)	2433 (2.77)	8682 (2.3)	<0.001
	*Pseudomonas aeruginosa*, *n* (%)	485 (1.13)	675 (1.36)	912 (1.58)	1253 (1.94)	1610 (2.15)	1731 (1.97)	6666 (1.77)	<0.001

T2DM: Type 2 Diabetes Mellitus. *p*-values < 0.05 to assess the time trend from 2001 to 2018. Using the χ^2^ test for the linear trend (proportions). According to the Bonferroni correction for multiple comparisons, the critical value (alpha) should be divided by 6 (0.05/6 = 0.0083).

**Table 4:** Variables associated with in-hospital mortality in hospital admissions of T2DM patients with a principal diagnosis of urinary tract infection according to sex in Spain (2001–2018).

Variables	Men	Women	Both
Female sex	NA	NA	1.1 (1.05–1.14)
18–50 years	1	1	1
51–64 years	1.55 (0.93–2.57)	1.83 (1.14–2.93)	1.72 (1.22–2.43)
65–74 years	3.11 (1.91–5.08)	3.74 (2.38–5.87)	3.49 (2.5–4.86)
75–84 years	5.3 (3.26–8.61)	7.3 (4.67–11.39)	6.37 (4.59–8.85)
≥85 years	9.8 (6.03–15.94)	12.8 (8.2–19.97)	11.41 (8.22–15.85)
CCI = 0	1	1	1
CCI 1–2	1.71 (1.56–1.87)	1.58 (1.48–1.69)	1.62 (1.54–1.71)
CCI > 2	2.72 (2.48–2.98)	2.28 (2.13–2.44)	2.44 (2.31–2.58)
Urinary catheter	0.69 (0.6–0.78)	1.2 (1.07–1.36)	0.91 (0.83–0.99)
*Staphylococcus aureus*	1.4 (1.19–1.66)	1.82 (1.57–2.13)	1.62 (1.45–1.81)
*Klebsiella pneumoniae*	0.69 (0.59–0.81)	0.75 (0.67–0.84)	0.73 (0.66–0.8)
*Escherichia coli*	0.5 (0.45–0.55)	0.45 (0.42–0.48)	0.46 (0.44–0.49)
Year	0.89 (0.87–0.91)	0.93 (0.91–0.94)	0.91 (0.9–0.93)

T2DM: Type 2 Diabetes Mellitus. NA: Not adequate. CCI: Charlson Comorbidity Index. Only variables included in the final model are shown in the table. Age, sex, and CCI are confounders. No significant interactions were found. Time (year) is included as a continuous variable.

## Supplementary Materials

The following are available online at https://www.mdpi.com/1660-4601/17/24/9427/s1: Table S1: ICD-9-CM and ICD-10 codes for the clinical diagnosis and procedures used in this investigation and Table S2: Variables associated with in-hospital mortality in hospital admissions of patients with a principal diagnosis of urinary tract infection according to sex in Spain (2001–2018).
