# Supplementary material for: Time Trends in Spain from 2001 to 2018 in the Incidence and Outcomes of Hospitalization for Urinary Tract Infections in Patients with Type 2 Diabetes Mellitus
Source: Int J Environ Res Public Health. 2020 Dec 16;17(24):9427. doi: 10.3390/ijerph17249427 (PMC7765668; doi:10.3390/ijerph17249427)
Supplement: Supplementary file 1 [file ijerph-17-09427-s001.pdf]

Supplementary Table S1. ICD 9 MC and ICD 10 MC codes used to identify diagnosis and procedures used in this investigation.

DIAGNOSIS/PROCEDURES	ICD 9 CM	ICD 10 CM
Enterococcus	041.04	B95.2; A40.2; A41.81
Staphylococcus aureus	041.1; 041.10; 041.11; 041.12; 041.19; 038.1; 038.10; 038.11; 038.12; 038.19	B95.6; B95.61; B95.62; B95.7; B95.8; A41.0; A41.1; A41.2 A41.01; A41.02
Klebsiella pneumoniae	041.3	B96.1
Escherichia coli	**041.4; 041.41; 041.42; 041.43; 041.49; 038.42**	**B96.21; B96.22; B96.23; B96.20 B96.29; A41.51**
Proteus (mirabilis) (morganii)	041.6	B96.4
Pseudomonas aeruginosa	041.7; 038.43	B96.5; A41.52
Urinary catheter	57.94; 57.95	0T9B70Z; 0T9B80Z; 0T2BX0Z

Supplementary Table S2. Variables associated with in-hospital mortality in hospital admissions of patients with a principal diagnosis of urinary tract infection according to sex in Spain (2001-2018).

	**Men**	**Women**	**Both**
Female sex	NA	NA	1.06(1.04-1.08)
<50 years	1	1	1
50-64 years	2.27(1.95-2.66)	5.84(5-6.81)	4.21(3.77-4.7)
65-74 years	4.25(3.68-4.92)	11.73(10.15-13.54)	8.14(7.35-9.02)
75-84 years	7.68(6.67-8.84)	22.95(20-26.34)	15.29(13.85-16.87)
>85 years	14.29(12.41-16.45)	41.25(35.97-47.29)	27.92(25.31-30.8)
CCI =0	1	1	1
CCI 1-2	1.76(1.68-1.84)	1.64(1.59-1.7)	1.69(1.65-1.74)
CCI>2	2.59(2.47-2.71)	2.31(2.23-2.41)	2.43(2.36-2.51)
Urinary catheter	0.71(0.66-0.76)	1.13(1.05-1.21)	0.87(0.83-0.92)
Staphylococcus aureus	1.47(1.35-1.61)	1.74(1.59-1.9)	1.59(1.5-1.7)
Klebsiella pneumoniae	0.75(0.68-0.81)	0.78(0.72-0.83)	0.77(0.73-0.81)
Escherichia coli	0.52(0.49-0.54)	0.47(0.45-0.49)	0.49(0.47-0.5)
Pseudomonas aeruginosa	0.75(0.7-0.81)	0.82(0.75-0.9)	0.86(0.81-0.91)
Year	0.92(0.91-0.93)	0.94(0.94-0.95)	0.93(0.93-0.94)
Diabetes	0.95(0.90-1.02)	0.99(0.93-1.04)	0.97(0.91-1.01)
